
JOURNAL INFORMATION
==============================
NLM Title Abbreviation: Anim Front
Journal ID: Anim Front
NLM Journal ID: 101754918
Journal ID: af

Animal Frontiers: The Review Magazine of Animal Agriculture

ISSN: 2160-6056
EISSN: 2160-6064
Publisher: Oxford University Press

ARTICLE INFORMATION
==============================
PMCID: PMC6951967
PMID: 32002276
DOI: 10.1093/af/vfz040
Article ID: vfz040
Article version: 1
Subjects: Society News

News from the Canadian Society of Animal Science

Publication date: 2019 Oct
Electronic publication date: 2019 Sep 28
Volume: 9
Issue: 4
First page: 60
Last page: 60
Copyright: Copyright © 2019 American Society of Animal Science
Copyright year: 2019
License: This is an Open Access article distributed under the terms of the Creative Commons Attribution License (http://creativecommons.org/licenses/by/4.0/), which permits unrestricted reuse, distribution, and reproduction in any medium, provided the original work is properly cited.
License URL: https://creativecommons.org/licenses/by/4.0/

Page count: 1

==============================
### News from the Canadian Society of Animal Science

The 2018 joint annual meeting of the American and Canadian Societies of Animal Science took place in Austin, Texas in July this year. Canadians were well-represented with two Symposia featuring Canadian content, graduate student competitions, and participation of many scientists. We extend our gratitude to all our sponsors, in particular generous support from Canadian Science Publishing, Diamond V, Trouw Nutrition, Masterfeeds LLP, Dairy Farmers of Canada, and the Canadian Pork Council.

Canadian Society of Animal Science | Société canadienne de Science animale
www.csas.net

Our 2019-2020 executive committee was also named at the Annual Meeting, and we look forwarding to serving our society in the coming year! We also warmly welcome our new Student Ambassadors, Michaela Lièvre, University of Guelph and Cameron Olsen, University of Alberta, who will represent the students of Eastern and Western Canada, respectively, on our Executive board.

### Graduate Student Awards at the 2019 Annual Meeting in Austin

The annual meeting provides an opportunity to recognize excellent science being done in Canada. Both students and professionals were recognized for their achievements with a number of CSAS Awards presented at the 2019 CSAS Awards Banquet. Student awards are sponsored by Canadian Science Publishing.

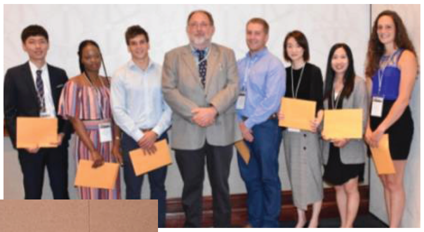

### Graduate Student Travel Fellowship Winners:

**Qianru Hui**, University of Manitoba; **Cameron Olsen**, University of Alberta; **Xuechun Bai**, University of Alberta; **James Templeman**, University of Guelph; **Janghan Choi**, University of Manitoba; **Prince Opoku**, University of Alberta; **Stèphanie Bèlanger-Naud**, McGill University; **Esther Ijiwad**, University of Alberta

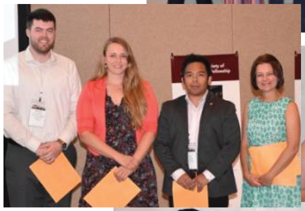

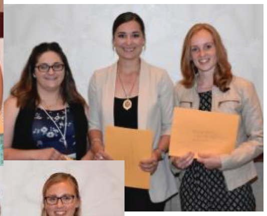

### Graduate Student Poster Competition Winners:

1st: **Ryley Vanderhout**, University of Guelph;
2nd: **Anouk Caron**, Université Laval;
3rd: **Paul Tamayao**, University of Manitoba

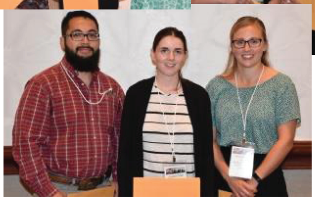

### MSc Oral Presentation Award Winners:

1st: **Annemiek Welborn**, University of Guelph;
2nd: **Nicole Briggs**, University of Alberta;
3rd: **Dakota Wightman** – University of Saskatchewan

### PhD Oral Presentation Award Winners:

1st: **Christine Bone**, University of Guelph;
2nd: **Stephanie Terry**, AAFC Lethbridge / University of Sydney;
3rd: **Daniel Watanabe**, University of Saskatchewan

### Professional Member Awards

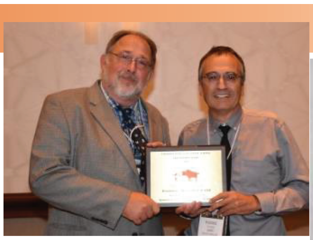

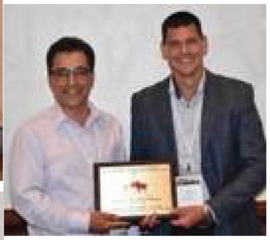

### Canadian Society of Animal Science Fellowship:

**Dr. Pierre Lacasse** *Agriculture and Agri-Food Canada;*
Sponsored by Canadian Science Publishing

### Technical Innovation in Enhancing Production of Safe and Affordable Food:

**Dr. Trevor Devries** *University of Guelph*;
Sponsored by Dairy Farmers of Canada, Canadian Pork Council, and The Canadian Society of Animal Science

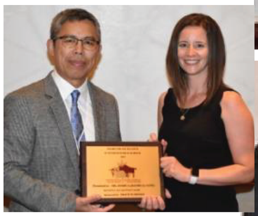

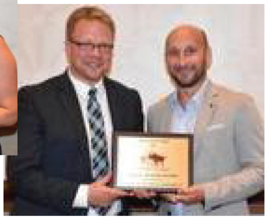

### Excellence in Nutrition and Meat Sciences:

**Dr. Joshua Gong** Agriculture and Agri-Food Canada;
Sponsored by Trouw Nutrition

### Young Scientist Award:

**Dr. Daniel Columbus** Prairie Swine Centre;
Sponsored by the employees of Masterfeeds LLP

### Join us at www.asas.org.CSAS!

doi: 10.1093/af/vfz040
